# Molecular Basis and Therapeutic Strategies to Rescue Factor IX Variants That Affect Splicing and Protein Function

**DOI:** 10.1371/journal.pgen.1006082

**Published:** 2016-05-26

**Authors:** Mojca Tajnik, Malgorzata Ewa Rogalska, Erica Bussani, Elena Barbon, Dario Balestra, Mirko Pinotti, Franco Pagani

**Affiliations:** 1 Human Molecular Genetics, International Centre for Genetic Engineering and Biotechnology, Trieste, Italy; 2 Department of Life Sciences and Biotechnology, University of Ferrara, Ferrara, Italy; University of Southampton and Wessex Clinical Genetics, UNITED KINGDOM

## Abstract

Mutations that result in amino acid changes can affect both pre-mRNA splicing and protein function. Understanding the combined effect is essential for correct diagnosis and for establishing the most appropriate therapeutic strategy at the molecular level. We have identified a series of disease-causing splicing mutations in coagulation factor IX (FIX) exon 5 that are completely recovered by a modified U1snRNP particle, through an SRSF2-dependent enhancement mechanism. We discovered that synonymous mutations and missense substitutions associated to a partial FIX secretion defect represent targets for this therapy as the resulting spliced-corrected proteins maintains normal FIX coagulant specific activity. Thus, splicing and protein alterations contribute to define at the molecular level the disease-causing effect of a number of exonic mutations in coagulation FIX exon 5. In addition, our results have a significant impact in the development of splicing-switching therapies in particular for mutations that affect both splicing and protein function where increasing the amount of a correctly spliced protein can circumvent the basic functional defects.

## Author Summary

Clarification of if an exonic variant has an effect on splicing and/or on protein function is an important aspect in clinical genetics and in development of appropriate therapeutic strategies, and most of published evidence consider splicing and protein function separately. In exons, the presence of dense splicing regulatory and amino acidic coding information implies that mutations may have a double pathogenic effect acting on splicing and/or on protein function. To address this issue we focused on coagulation factor IX (FIX) exon 5, where we identified natural mutations that induce different degree of exon skipping. All exon skipping mutations were completely corrected by a novel splicing-switching therapeutic approach based on modified U1 snRNP. To detect the substitutions that might benefit from this correction, we investigated splicing recovered mutations for FIX protein secretion and specific activity. This analysis identified synonymous mutations causing remarkable exon skipping and missense mutations with a partial effects on both splicing and secretion, but compatible with normal FIX coagulant properties, as target variants for the splicing-switching therapy.

## Introduction

Exonic mutations represent by far the most frequent cause of human genetic disorders,[1] and their pathogenic effect is usually attributed to alterations of the amino acid code. However, the exons contain also an intricate series of splicing regulatory elements (“splicing code”) that are essential for their recognition and overlap with the aminoacid code. In fact, the correct selection of canonical splice sites that define the exon boundaries (3’ss and 5’ss, respectively, which include the polypyrimidine tract and the branch site at the 3’ss) requires a series of auxiliary regulatory elements. According to their location and activity, the auxiliary elements are known to function as exon splicing enhancer and silencers (ESEs and ESEs, respectively) or intron splicing silencers (ISSs and ISSs, respectively). Typically, in the exon, Serine/arginine-rich (SR) proteins[2] recognize ESE whereas heterogenous nuclear RiboNucleoProteins (hnRNP) interact with ESS,[3] inducing exon inclusion or skipping, respectively. Due to the presence of these regulatory elements, exonic mutations are strong candidates to affect splicing and the most frequent defect they produce is exon skipping, as shown in several disease genes and model systems.[4–8] However, in affected genes, the relative contribution of splicing and protein function in the disease pathogenesis is largely unknown. Intriguingly, while correction strategies for missense mutations are far from being proved, there are several tools enabling the rescue of splicing and proposing them as innovative therapeutic strategy, which include antisense oligonucleotides, chemical compounds and modified U1 small nuclear RNAs (U1 snRNA).[9–12] Moreover, these strategies can be exploited in combination.[13] In exon skipping mutations, modification of the U1 snRNA, which is the key component of the spliceosomal small nuclear ribonucleoprotein (U1 snRNP), is able to rescue exon skipping. The engineered U1 snRNPs defined as Exon Specific U1s (ExSpeU1) bind in the intron downstream of the 5’ splice site and rescue exon skipping variants in Spinal Muscular Athrophy[14], Netherton syndrome[15] Cystic fibrosis and Hemophilia B.[9] The activity of these molecules in primary cells derived from patients[14, 15] and *in vivo* in mouse models through AAV delivery[14] suggests that ExSpeU1s have a strong therapeutic potential. In factor IX (*FIX*) exon 5, ExSpeU1s rescued exon-skipping mutations at the 5'ss and at the polypyrimidine tract with a complete recover of the functional factor IX activity.[9] Mutations is *FIX* exon 5 are associated to Hemophilia B, a rare X-linked hemorrhagic disorder (1/35000 males) with reduced levels of factor FIX, a key coagulation protein of liver origin.[16] The level of FIX antigen or clotting activity in the plasma determines the variability of the disease severity. [17] Hemophilia B represents a paradigmatic example of human disease with a heterogeneous mutational pattern [18] and even a small increase of FIX levels (>2%) would significantly ameliorate the clinical phenotype. The FIX gene contains eight exons and seven introns and transcribe 2.8 kb long mRNA [19].

Exonic mutations (missense, nonsense and synonymous) represent the first cause of coagulation factor deficiencies, thus providing ideal models to address the relationship between splicing and protein function. As a matter of fact, in several cases the results from the expression of the missense coagulation factor variants did not recapitulate the residual expression levels in the affected patients and disease causing mechanism of synonymous mutations is frequently unclear. In this study, we focused on exonic mutations in FIX gene that have been found in Hemophilia B patients, and particularly on exon 5 that encodes for EGF2 domain, which is crucial for the coagulant activity in the intrinsic coagulation pathway.[20] This exon contains several missense, nonsense and synonymous mutations,[21] whose potential effect on pre mRNA splicing has not been studied so far.

Here, we show for the first time that *FIX* exon 5 contains dense splicing regulatory information that overlap with the aminoacid code and that several *FIX* exonic mutations result in exon skipping. Furthermore, a unique ExSpeU1, through an *SRSF2-*mediated mechanism, rescues all *FIX* splicing defects. Most importantly, through complementary expression studies with minigene splicing assays and full-length protein constructs we dissected for each mutation the relative contribution of splicing alteration, defective protein secretion or abnormal coagulant activity. Lastly, this relationship allows us to select those exonic mutations that most likely will have a therapeutic benefit from splicing correction.

## Results

### FIX exon 5 contains dense splicing regulatory information

**Fig 1:**
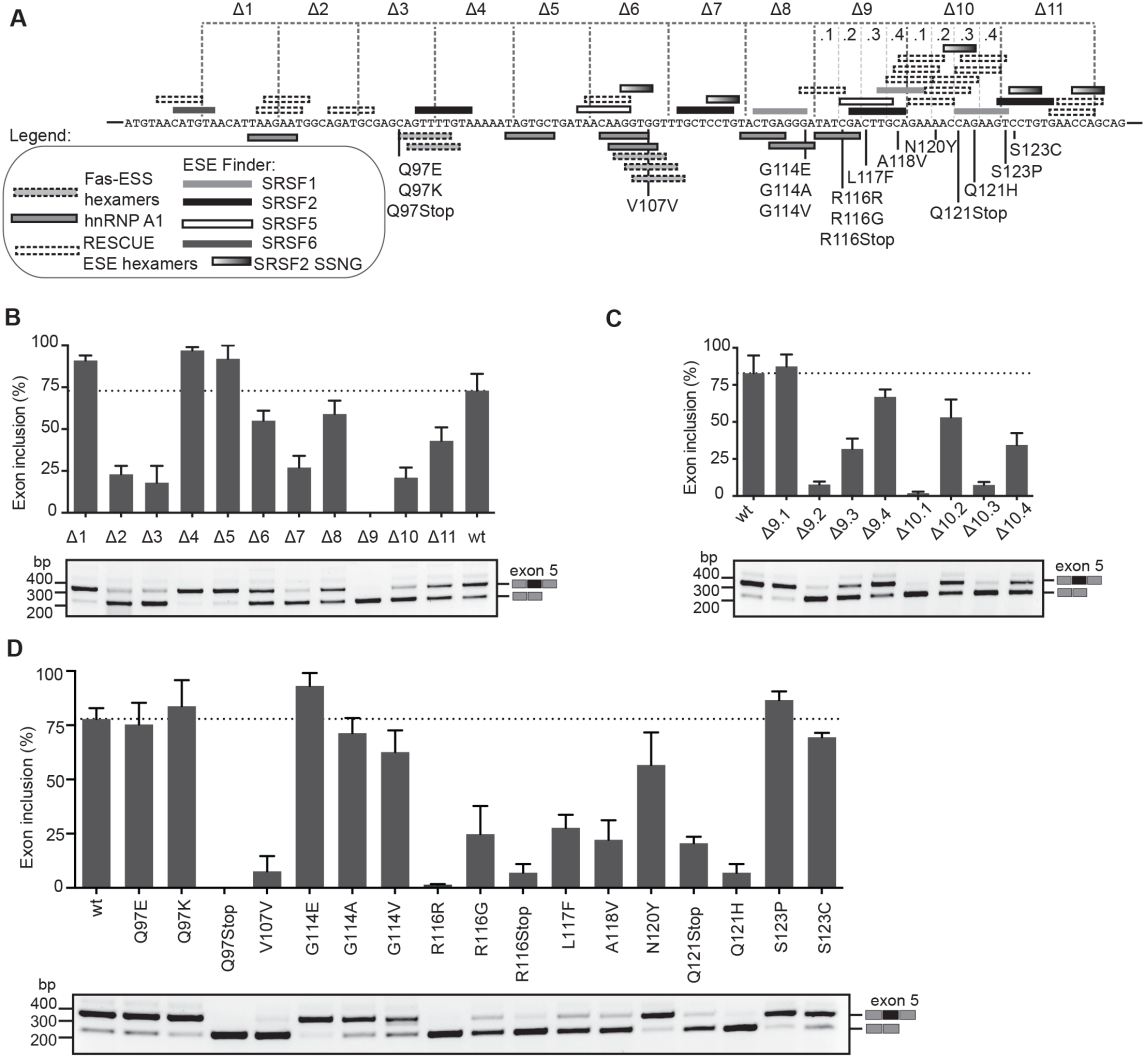
Identification of splicing regulatory elements and exon skipping mutations in FIX exon 5. (A) Schematic representation of FIX exon 5 sequence showing the location of the deletions, the exonic mutations and *in silico* predicted binding sites for splicing factors. Nucleotide changes for exonic mutants are shown in Table 1. (B) Splicing assays of minigenes carrying the 10bp deletions (Δ1 to Δ11). Minigenes were transfected in HeLa cells and the splicing pattern evaluated by RT-PCR. The levels of exon inclusion were quantified by ImageJ and compared to the wild-type. The identity of the amplified bands with inclusion or exclusion of exon 5 is indicated (C) Splicing assays of minigenes with 3 bp deletions (Δ9.1 to Δ10.4) (D) Minigene splicing assay of disease-causing exonic mutations. Data in all panels are the average of 3 replicates with error bars indicating SD.

To map exonic splicing regulatory elements in FIX exon 5 we initially performed multiple deletions analysis. We created eleven 10 bp-long deletions distributed throughout the entire exon (from Δ1 to Δ11) (Fig 1A). These deletions were tested in the previously validated FIX exon 5 minigene system[9], where the WT exon is not completely defined, showing ~80% of exon inclusion as reported in human liver [9]. Consistent with the presence in the exon of dense splicing regulatory information, most deletions affected splicing. Based on the splicing changes, we identified two (Δ4 and Δ5) and eight (Δ2, Δ3, Δ6, Δ7, Δ8, Δ9 and Δ10) regions with silencer and enhancer properties, as their deletion resulted in exon inclusion or skipping, respectively (Fig 1B). As the most striking and deleterious effect on splicing was observed with the Δ9 deletion and neighboring Δ10 deletion, we evaluated more in detail these regions by creating 3 bp long deletions. Splicing assays showed that Δ9.2, Δ10.1 and Δ10.3 induced significant exon skipping (<10%) (Fig 1C), which suggests the presence of multiple and overlapping exonic regulatory sequences in this region. To further clarify the role of exonic elements, we mapped binding sites of SR-proteins according to ESE finder,[22] hnRNPA1 motifs[23] and predicted ESE and ESS [24, 25]. *In silico* analysis showed the presence in the exon of multiple and frequently overlapping silencers and enhancers (Fig 1A). Interestingly, the Δ9 and Δ10 regions, associated to severe exon skipping, contain several potential ESEs. In addition, among the SR proteins and according to ESE finder, SRSF2 is the most represented factor with four potential binding sites. Moreover, the exon contains also 5 recently identified consensus SRSF2 binding motif SSNG[26] (S = C/G, N = any) (Fig 1A). Thus, both *in silico* and experimental data indicate that FIX exon 5 contains several splicing regulatory elements with both enhancer and silencer function. The presence of these dense splicing regulatory elements suggests that FIX exon 5 may be extremely susceptible to mutation-induced splicing derangement.

### Synonymous, missense and nonsense disease-causing mutations affect FIX exon 5 recognition

The FIX mutation database reports several missense, nonsense or even synonymous mutations in exon 5 that are associated with Hemophilia B.[21] To understand the contribution of exonic mutations on splicing, we focused on changes with unclear disease-causing mechanism (synonymous or conservative amino acid substitutions) and/or on variants located in strongest splicing regulatory elements (i.e. in the Δ9 and Δ10 elements). Overall, we evaluated 16 disease-causing mutations: 2 synonymous (V107V and R116R), 11 missense and 3 nonsense (Table 1). We also include in the analysis the artificial R116G variant. Strikingly, in splicing assay, 9 mutations induced significant exon 5 skipping (Fig 1D and Table 1). In this experimental system, R116R and Q97Stop showed complete or nearly complete exon skipping (below 5% of exon inclusion) suggesting that in these cases the primary disease-causing mechanism is the severe splicing defect. Interestingly, 6 mutations maintained some residual levels of normal splicing: V170V, R116Stop and Q121H showed between 5–10% of exon inclusion whereas R116G, L117F, A118V and Q121Stop showed ~ 25% of exon inclusion. The presence of some degree of leaky splicing indicates that in these cases the splicing defect contributes partially to the disease pathogenesis. These results clearly indicate that a significant proportion of exon 5 mutations negatively affect the splicing process.

**Table 1:** Mutations in FIX exon 5.

Mutation	Nucleotide number	Nucleotide change	Patient FIX:Ag	Patient FIX:C	Severity	Exon inclusion (%)	cDNA FIX:A (%)	cDNA FIX:C # (%)	ESM class^
**Q97Stop**	17704	C>T	n/a	n/a	Severe	2.4	n/a	n/a	n/a
**R116R**	17761	C>A	5–7	3–6	Mild/Moderate	2.0	96±5	96±8	I
**Q121H**	17778	G>T	n/a	18	Mild	7.0	26±3	110±15	II
**R116Stop**	17761	C>T	<1–3	<1–1	Severe	7.0	n/a	n/a	n/a
**V107V**	17736	G>A	14–24	12–21	Mild	7.6	100±6	81±22	I
**Q121Stop**	17776	C>T	<1	<1	Severe	20.6	n/a	n/a	n/a
**A118V**	17768	C>T	n/a	25	Mild	22.2	17±1.6	92±7	II
*R116G	17761	C>G	n/a	n/a	n/a*	24.8	27±2	88±15	II
**L117F**	17764	C>T	n/a	<1	Severe	27.7	0	0	III
**N120Y**	17773	A>T	0.4	<1	Severe	56.7	n/a	n/a	n/a
**G114V**	17756	G>T	2	<1–1	Moderate/Severe	62.6	n/a	n/a	n/a
**S123C**	17783	C>G	n/a	15	Mild	69.5	n/a	n/a	n/a
**G114A**	17756	G>C	4–5	5–13	Mild/Moderate	71.3	n/a	n/a	n/a
**Q97E**	17704	C>G	<1	<1	Severe	75.3	63.5±5.8	5.9±2	n/a
**Q97K**	17704	C>A	n/a	<1	Severe	83.8	60±0.2	2.9±1	n/a
**S123P**	17782	T>C	n/a	<1	Severe	86.6	n/a	n/a	n/a
**G114E**	17756	G>A	8	4–7	Mild/Moderate	93.1	n/a	n/a	n/a

^#^ specific activity, n/a not applicable,

*artificial mutant. Patient FIX:Ag, FIX:C and severity are from FIX database.

^Exonic Splicign Mutations (ESM) classes are listed according to Fig 5.

** cDNA FIX:A and cDNA FIX:C are expressed as percentage of WT FIX activities

### SRSF2 is a crucial factor regulating FIX exon 5 inclusion

**Fig 2:**
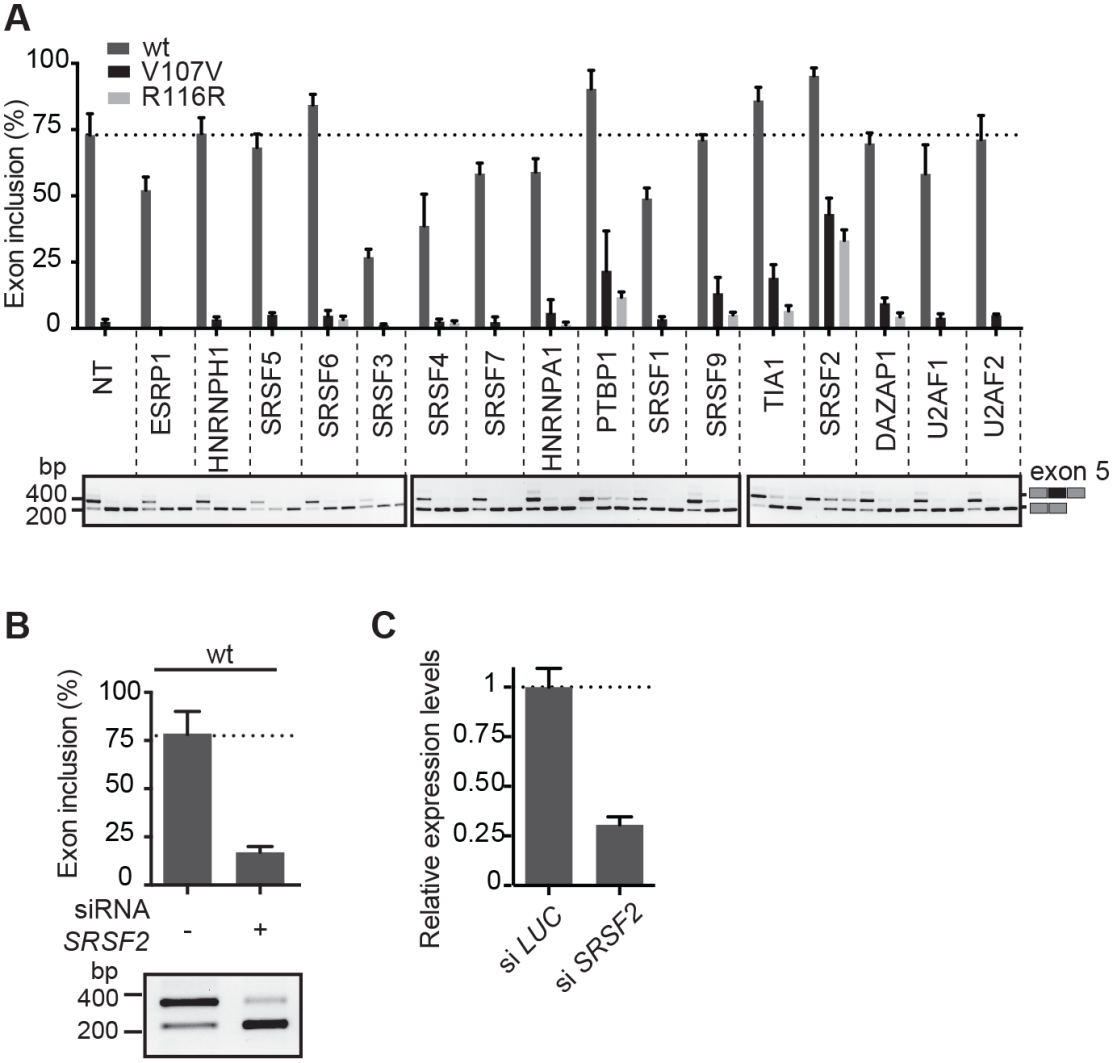
Identification of SRSF2 as the main splicing factor responsible for FIX exon 5 recognition. (A) Effect of overexpression of a panel of splicing factors on the splicing pattern in the FIX exon 5 wild-type and mutant minigenes (V107V and R116R). Minigenes were transfected in HeLa cells alone (NT) or with the indicated splicing factors and the splicing pattern evaluated by RT-PCR. The identity of the amplified bands with inclusion or exclusion of exon 5 is indicated. (B) Silencing of SRSF2 reduces the percentage of exon 5 inclusion in wild-type minigene. siRNA treated (+) and control HeLa cells (-) were transfected with wt minigenes and the resulting splicing pattern evaluated by RT-PCR. (C) Relative expression level of SRSF2 RNA, analyzed by qRT-PCR in siSRSF2 and control (si Luc) Hela cells. All data in the three panels represent the average of 3 replicates with error bars indicating SD.

To identify splicing factors potentially involved in regulating exon 5 splicing, we performed overexpression experiments. Wild-type (wt) and two mutated minigenes (V107V and R116R) were co-transfected with different splicing factors followed by analysis of the splicing pattern (Fig 2A). Several factors including most of the Serine/arginine-rich proteins (SRSF3, SRSF4, SRSF7 and SRSF1) along with ESRP1 and hnRNP A1 had a negative effect on splicing, reducing the percentage of exon inclusion in the wild-type minigene. In contrast, Polypyrimidine tract binding protein 1 (PTBP1), Cytotoxic granule-associated RNA binding protein (TIA1), and Serine/arginine-rich splicing factor 2 (SRSF2) induced exon inclusion. This enhancing effect was also evident for the two synonymous exonic variants. Since SRSF2 was the most active factor, and the only serine/arginine-rich splicing factor with enhancing activity on exon 5, we evaluated more in detail its role through silencing experiments. Indeed, in the wt context, SRSF2 silencing reduced the percentage of exon 5 inclusion, strongly suggesting that this factor is crucial for its definition (Fig 2B and 2C). In parallel, to understand the potential regulatory sequences that mediate the SRSF2-dependent splicing enhancement, we tested the effect of SRSF2 overexpression on the deletion mutants (S1 Fig). The transfection experiments showed that SRSF2 significantly improves exon skipping in almost all deletion mutants, suggesting that this factor promotes exon 5 definition through its binding throughout the entire exon.

### Splicing rescue of exonic mutations by ExSpeU1 requires SRSF2

**Fig 3:**
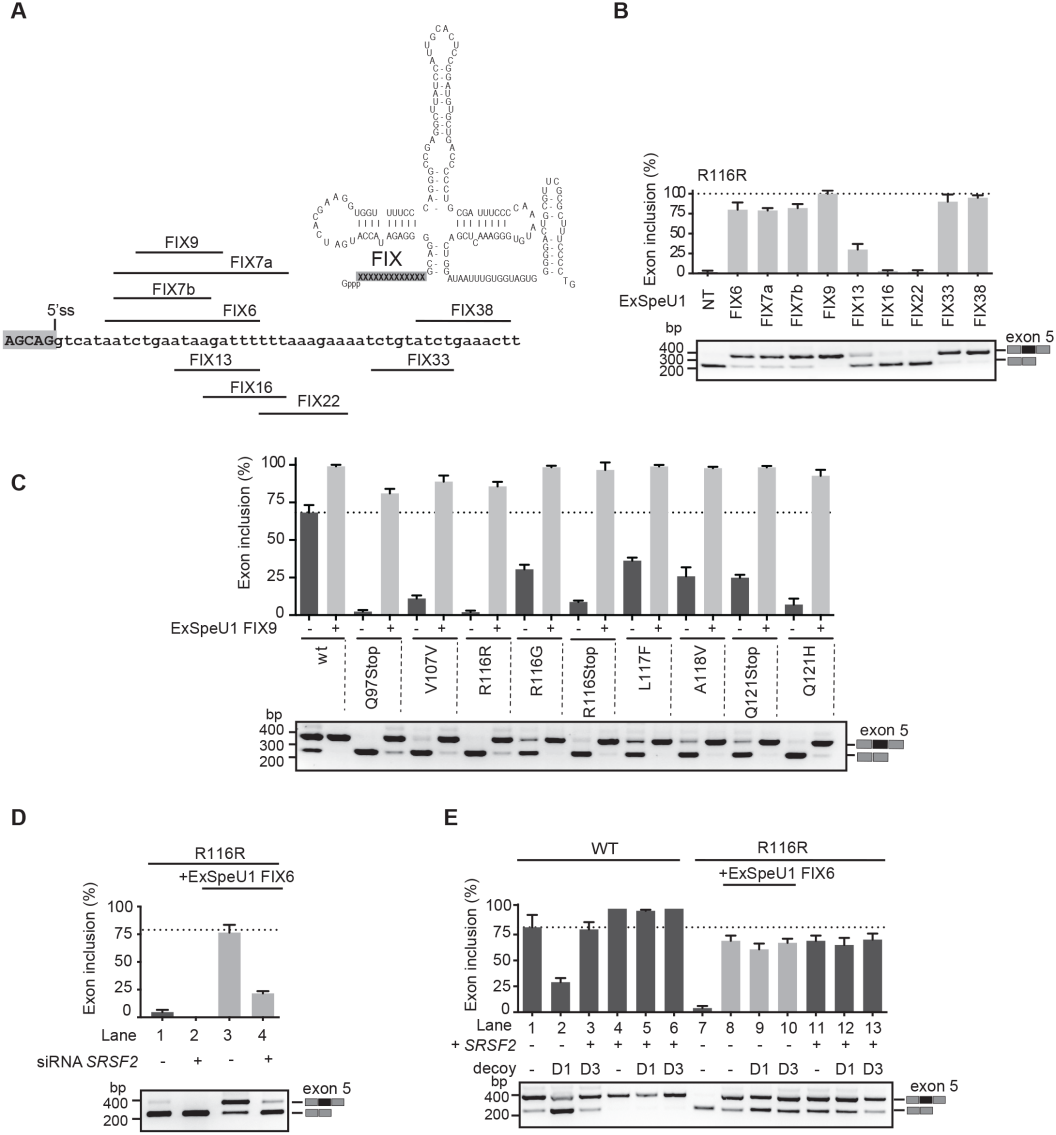
ExSpeU1 rescue exonic mutations in FIX exon 5 through an *SRSF2*-mediated mechanism. (A) Schematic representation of the secondary structure of ExSpeU1 RNA highlighting the 5' tail region (FIX). FIX exon 5 (uppercase) and intron 6 (lowercase) sequences are indicated along with the ExSpeU1 binding regions. (B) Cotransfection experiments. The R116R minigene was transfected in HeLa cells alone (NT) or with the indicated ExSpeU1s and the resulting splicing pattern evaluated by RT-PCR. (C) Splicing rescue activity mediated by ExSpeU1 FIX9 on exon skipping mutations. The indicated minigenes were co-transfected with (+) or without (-) ExSpeU1 FIX9 and the pattern of splicing evaluated. All data are the average of 3 replicates with error bars indicating SD. (D) ExSpeU1 rescue depends on SRSF2. siRNA treated (+) and control HeLa cells (-) were transfected with R116R minigene in the presence (lanes 3 and 4) or absence (lanes 1 and 2) of ExSpeU1. Data represent the average of 3 replicates with error bars indicating SD. (E) *SRSF2* and ExSpeU1 act regardless of endogenous U1snRNP. Wt and R116R minigene were transfected alone (lane 1 and 7, respectively) or in the presence of the indicated plasmids. D1 is antisense to the endogenous snRNA, whereas D3 is the control that contains a mutation that prevents binding to U1. In all panels data are the average of 3 replicates with error bars indicating SD.

We previously reported that modified U1 snRNA binding in *FIX* intron 5 rescued exon skipping caused by mutations located at the polypyrimidine tract or at the 5'ss.[9] To evaluate the potential therapeutic effect of ExSpeU1s on the exonic mutations, we initially tested a panel of ExSpeU1s that bind at different intronic positions (Fig 3A). These molecules were evaluated on R116R that, by remarkably affecting exon definition (Fig 1D), is one of most severe exonic mutation identified. Out of 9 ExSpeU1 FIX tested, 6 rescued exon 5 inclusion at least to the level of the wild-type condition (Fig 3B). The lower or absent rescue activity in FIX13, FIX16 and FIX 22 ExSpeU1s could be due either to their lack of interaction with the corresponding intronic sequences or to their reduced expression levels. One of the active ExSpeU1s was then evaluated on the natural splicing mutations (Fig 3C) and on the artificial deletion mutants (S2 Fig). The splicing assays showed that cotransfection of ExSpeU1 completely rescued all exon-skipping events. Therefore, loading of modified U1 snRNA on FIX intron 5 represents a therapeutic strategy for splicing correction not only for polypyrimidine and 5'ss mutations[9] but also for mutations that affect splicing regulatory elements located in the exon. To address whether the ExSpeU1-mediated splicing enhancement requires *SRSF2*, we performed silencing experiments. The SRSF2-silenced cells were cotransfected with ExSpeU1 and R116R variant. As expected, ExSpeU1 completely restored exon inclusion, but the concomitant silencing of SRSF2 remarkably reduced its activity (Fig 3D, compare lanes 3 with lane 4). In a second set of experiments, by exploiting U1 decoy molecules (Fig 3E), we evaluated whether SRSF2-mediated splicing improvement requires the endogenous U1. The U1 decoy D1 is an antisense RNAs that when trasfected in cells it functionally inhibits the normal U1 snRNP activity by complementarity[27]. Cotransfection of D1, but not the D3 control, was previously shown to induce exon skipping in several gene systems [14] [27]. As the ExSpeU1-mediated splicing enhancement on R116R is not appreciably affected by the U1 decoy (D1 treatment, Fig 3D, compare lanes 8 and 9), ExSpeU1 can functionally overcome the absence of the endogenous U1 snRNP. This result is consistent with previous data obtained in other gene systems.[14] When we tested the effect of the U1 decoy on the SRSF2-mediated splicing improvement, we observed that functional inhibition of the endogenous U1 with the D1 treatment has a minimal effect on the splicing pattern (Fig 3E, compare lanes 11 and 12). Similar result was obtained in the wild-type context where D1 treatment reduces splicing efficiency in WT minigene (Fig 3E, compare lanes 1 with 2) but has no effect in the presence of SRSF2 (Fig 3E, compare lanes 4 with 5). All together these results suggest that ExSpeU1 promotes splicing facilitating loading of SRSF2 on the defective FIX exon 5 sequences.

### V107V and R116R synonymous mutations create hnRNP A1 and DAZAP1 binding sites

**Fig 4:**
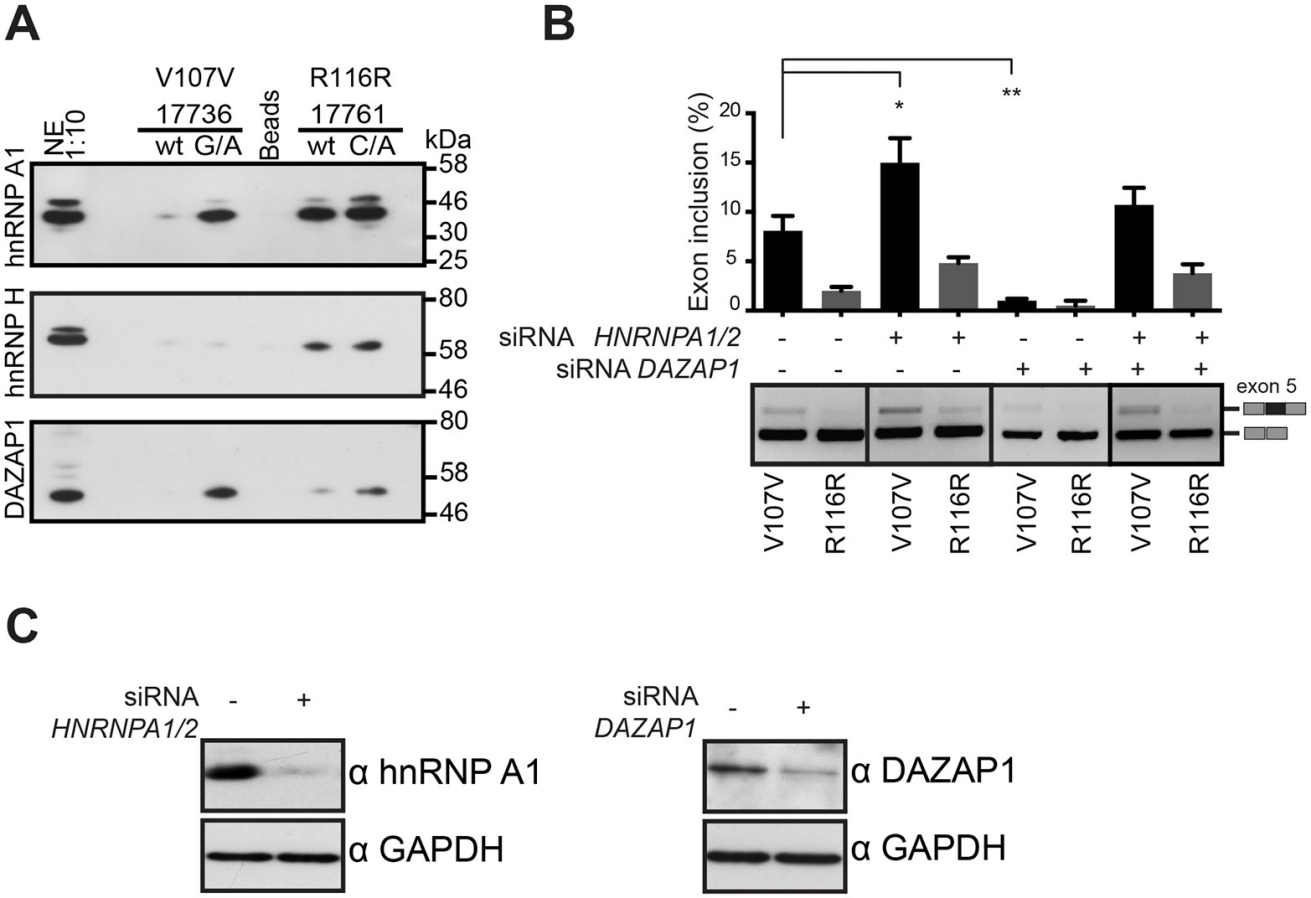
V197V and R116R mutant RNAs affect binding to inhibitory splicing factors. (A) Western blot analysis of hnRNP A1, hnRNP H and DAZAP1 proteins identified by pull-down mass-spectroscopy analysis. (B) FIX exon 5 inclusion levels upon silencing of *HNRNPA1/2* and *DAZAP1*, in V107V and R116R mutated minigenes. Data represent the average of 3 replicates with error bars indicating SD. Student *t*-test * p ≤ 0.05, ** p ≤ 0.01, *** p ≤ 0.001, *n*.*s*. not significant. (C) Efficiency of *HNRNPA1/2* and *DAZAP1* silencing analyzed by Western blot.

To understand how the exonic mutations negatively affect splicing we focused on the two splicing severe synonymous V107V and R116R variants, on which we performed protein pull-down experiments followed by mass-spectroscopy. This analysis identified three major splicing factors with splicing inhibitory activity that bind to the mutated sequences: DAZ associated protein 1 (DAZAP1), Heterogeneous nuclear ribonucleoprotein H1 (hnRNP H1) and Heterogeneous nuclear ribonucleoprotein A1 (hnRNP A1) (S3 Fig). Upon western blotting we confirmed increased hnRNPA1 and DAZAP1 binding on V107V and increased binding of DAZAP1 on R116R (Fig 4A). In contrast, hnRNP H1 did not show any differential binding between wt and mutant sequences. As hnRNP A1 is a well known splicing inhibitor [28–31], and DAZAP1 contributes to hnRNPA1 to exon skipping in another disease-causing mutation,[28] we evaluated their contribution with silencing experiments (Fig 4B). Silencing of *HNRNPA1/2* slightly increased the percentage of exon inclusion in V107V (from 8.1±1.5 to 15.0±2.5, *t*-test p≤0.05) whereas R116R was not appreciably affected. In contrast, *DAZAP1* did not promote exon inclusion and unexpectedly it reduced splicing in the V107V mutant (from 8.1±1.5 to 1.0±0.2, *t*-test p≤0.01) (Fig 4C). These data suggest that the formation of the novel binding sites for hnRNPA1 and DAZAP1 in the mutants partially contributes to exon skipping.

### Effect of the synonymous and missense mutations on FIX secretion and coagulant activity

**Fig 5:**
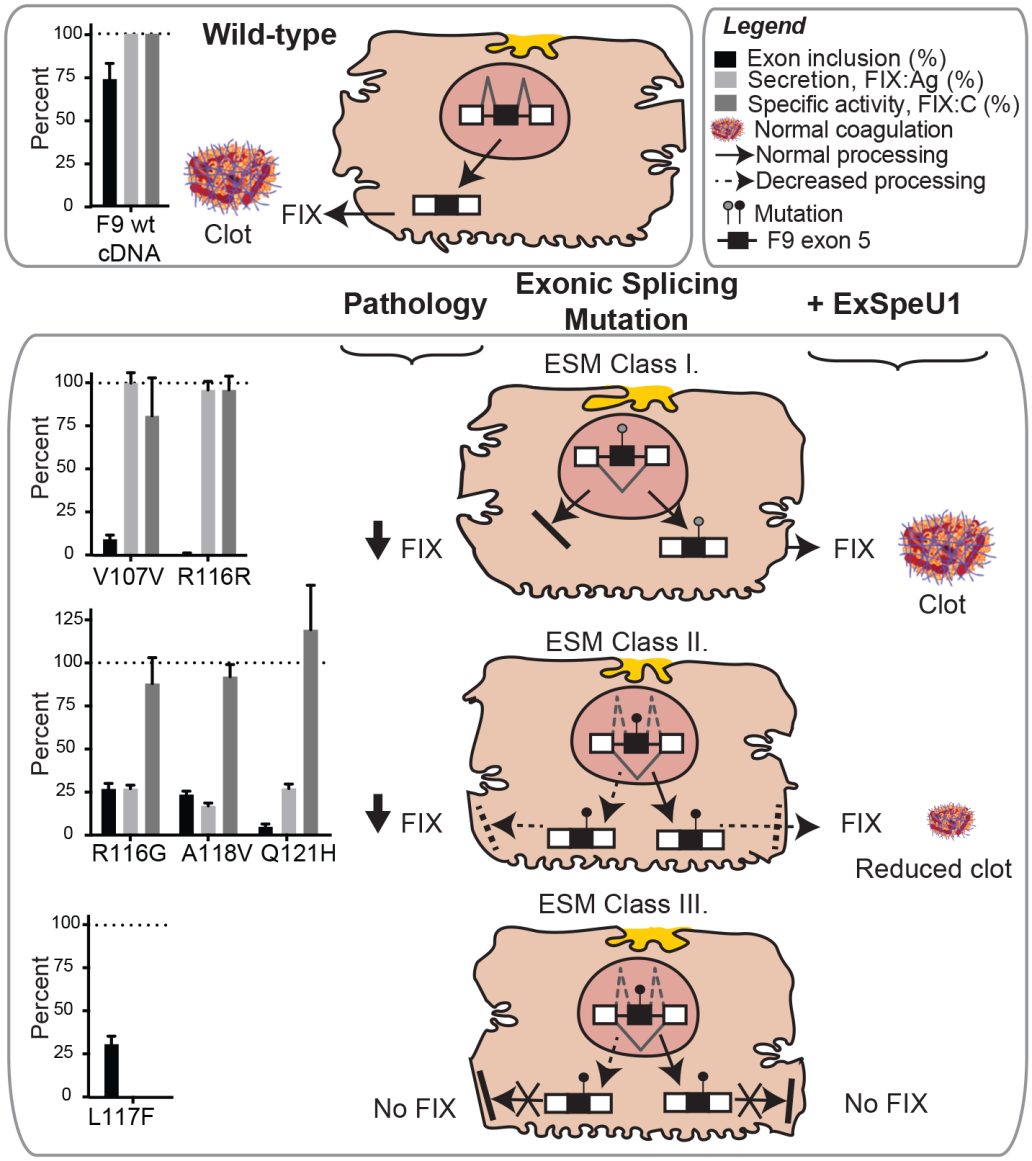
The effect of FIX exon 5 mutations on splicing and FIX secretion/activity explains the residual FIX levels in Hemophilia B patients and the therapeutic potential of a splicing-switching molecule. The upper box shows exon 5 splicing, secretion efficiency and specific activity levels of Factor IX in normal conditions. The lower box shows the three classes of Exonic Splicing Mutations (ESM) categorized according to their pathologic effect on exon 5 splicing, FIX secretion efficiency and FIX specific activity, and according to the therapeutic splicing rescue promoted by ExSpeU1. The graphs on the left of each class summarize the effect of each mutation on splicing, FIX secretion efficiency and FIX specific activity relative to the wild type as identified in this paper. Detail of the model are provided in discussion.

To explore those exon 5 mutations that could benefit from ExSpeU1- mediated splicing correction, we measured the effect of 2 synonymous and 6 missense mutations associated with exon skipping on the secreted FIX protein and coagulant activity levels (Table 1). Expression studies with the exonic variants in the FIX cDNA context, which is not influenced by splicing, showed that the synonymous V107V and R116R substitutions did not affect the protein biology nor influence or pause the ribosomal translation due to codon preferences[32] (Table 1, lanes cDNA FIX:Ag and cDNA FIX:C) (Fig 5). The R116G, A118V and Q121H missense mutations resulted in reduced secretion but strikingly, the lower amounts of the secreted proteins have a normal specific coagulant activity (Table 1, lanes cDNA FIX:Ag and cDNA FIX:C) (Fig 5). In contrast, L117F variant strongly impaired the FIX secretion, as indicated by the barely detectable FIX antigen in medium (Table 1)(Fig 5). Lastly, we included in the analysis as controls two mutations that do not affect splicing, Q97K and Q97E. These variants were efficiently secreted but displayed an impaired coagulant activity (Table 1).

## Discussion

Exons are known to accommodate two complementary and overlapping information: splicing signals and amino acids code. Exonic mutations are first checked by the spliceosome and then any residual amount of normally spliced transcript is evaluated for protein functionality. The final effect of a mutation on gene expression is the result of the severity of aberrant splicing along with the functional consequence of the amino acid substitution. In the paradigmatic model of FIX exon 5 we demonstrate that the final outcome, as well as the possible therapeutic rescue, depends on the impact of each mutation on mRNA splicing and/or protein biology. Our results clearly indicate that understanding the combined effect of exonic mutations on splicing and protein function is fundamental for correct diagnosis at the molecular level and for establishing the therapeutic feasibility of a splicing rescue strategy.

In normal conditions FIX exon 5 is correctly recognized by the spliceosome and mostly included in the final transcript. This leads to the production of a normal FIX protein that folds correctly in the ER, it is efficiently secreted and, when present in the blood, activates properly coagulation (Fig 5, WT). Based on the effect on splicing, secretion and coagulant activity, exonic splicing mutations (ESMs) can be divided into three major groups which define their molecular basis and potential therapeutic splicing intervention. In the first case, the disease-causing mechanism is entirely due to aberrant splicing and therefore splicing correction is therapeutic (Fig 5, class I). The two synonymous V107V and R116R variants belong to this class: they affect binding of splicing factors and induce severe exon skipping with the production of a non-functional mRNA. Their ExSpeU1-mediated splicing improvement result in the production of the correct mRNA and normal protein. Affected patients have mild/ moderate phenotype and the residual splicing levels well correlate with the reported FIX:ag and FIX:C activities (Table 1). In the second type of mutations (Fig 5, ESM class II), the presence of two defects, one on splicing and the other on secretion determines at the end the lack or very reduced amounts of the protein. In this case, the partial splicing defect produces low amounts of normally spliced transcript that, when translated, result in a defective FIX protein with a significantly reduced, but not completely abolished, secretion. However, as the protein once secreted maintains a normal coagulant activity, an efficient ExSpeU1-mediated correction is expected to partially rescue its function. Natural mutations A118V and Q121H, and the artificial R116G belong to this class (Fig 5, ESM class II); A118V and Q121H are associated to a mild phenotype and to residual FIX:C levels that well correlates with the presence of partial defects in splicing and secretion (Table 1). Lastly, mutations like L117F (Fig 5, ESM class III) show a splicing defect but, as the resulting amino acid change severely affects secretion, splicing improvement would not recover FIX function. In this case, splicing correction should be complemented by additional strategies to bypass the secretion defect and possibly improve misfolding. Interestingly, some nonsense mutations partially (Q121Stop) or severely (Q97Stop and R116Stop) affect splicing (Fig 1D) (Table 1). These nonsense ESMs might also indirectly benefit from ExSpeU1 splicing rescue. In the experimental system we have used, which is NMD-independent [33], the consequence of nonsense mutation on pre mRNA processing can be exclusively attributed to their effect on splicing. In these cases, the splicing correction will not have any direct positive effect by itself but become mandatory for subsequent read-through therapies.

In principle, exonic mutations can create a splicing silencer or disrupt a splicing enhancer. However, in several cases, due to the promiscuous composition of the regulatory elements and the intrinsic combinatorial control of splicing,[25, 34] both mechanisms are involved.[35, 36] Consistent with a major role of silencers in FIX exon 5, we show, using protein pull-down analysis coupled by mass-spectroscopy, that two synonymous mutations (V107V and R116R) increase binding to a negative splicing regulator hnRNP A1[28] and also to DAZAP1 (Fig 4A). DAZAP1 associate with hnRNPA1 and was previously implicated in exon skipping.[28] However, their silencing have a small effect on splicing (Fig 4C), suggesting that additional factors are involved. In any case, notwithstanding the exon skipping mechanism, ExSpeU1 rescued all nine exonic mutations (Fig 3B). Overall, including the previously reported five variants at the polypyrimidine tract and at the 5'ss,[9] a unique ExSpeU1 can rescue 14 different splicing mutations, increasing the number of affected individuals that would benefit from this therapeutic strategy. This effect on different types of mutations is probably due to the presence in the exon of several *SRSF2* binding sites. *SRSF2* is the most active factor promoting exon 5 inclusion (Fig 2) and its silencing inhibits the ExSpeU1-mediated splicing rescue (Fig 2C). Thus, binding of ExSpeU1 in the intron downstream the 5'ss facilitates recruitment of SRSF2 on the exon, compensating the negative effect of the exonic and intronic mutations. This mechanism is consistent with the fact that *SRSF2* is known to facilitate interaction between U1 and U2 snRNP.[37] The ExSpeU1 interaction with intronic sequences is also expected to reduce possible off targets with the advantage, in common with splicing correction strategies and in contrast to classical gene therapy approaches, of maintaining expression of the gene in the normal chromosomal context. In the perspective of a therapeutic intervention, AAV vector represents a reliable system to deliver ExSpeU1s, as we have recently demonstrated [14], and liver is a well established target tissue for this vector.[38]

In conclusion, our result establishes that mutations in FIX exon 5 can contribute to the disease combining splicing and protein dysfunctions and identifies those variants eligible for splicing-switching therapeutic molecules. In exons, dissection of the relative contribution of splicing versus amino acid dysfunction is critical for making a correct diagnosis at the molecular level and for establishing the therapeutic feasibility of a splicing rescue strategy. The splicing correction approach based on precise engineering of the U1 core spliceosomal RNP can be easily applied to different type of defective exons and diseases increasing the potential therapeutic spectrum of these novel class of molecules.

## Materials and Methods

### Creation of expression vectors

For the reporter minigene splicing assay, we have used the previously described pTBFIX exon 5 minigene.[9] Overlapping PCR approach was used for introducing disease-causing point mutations and oligonucleotides are listed in S1 Table. The minigenes of FIX exon 5 deletions (Δ1 to Δ11 and Δ9.1 to Δ10.4) were commercially synthesized (GenScript, NJ, USA). Expression vectors for the recombinant FIX variants were produced by site-directed mutagenesis using the QuickChange II Site-Directed Mutagenesis Kit (Stratagene, La Jolla, CA, USA). The mutations were introduced into the human FIX cDNA cloned into the pCMV5 vector[39], using primers listed in S1 Table. Exon-specific U1 snRNAs were created by replacing the sequence between the sites BclI and BglII with oligonucleotides as done previously.[9] Oligonucleotides are listed in S1 Table. All the clones were verified by sequencing.

### Cell cultures, transfection and FIX splicing and protein assays

Splicing Assays: HeLa cell line was grown in Dulbecco's modified Eagle's medium with Glutamax I (Gibco) (DMEM with glutamine, sodium pyruvate, pyridoxine and 4.5 g/l glucose) supplemented with 10% fetal calf serum (Euro Clone) and Antibiotic Antimycotic (Sigma). HeLa cells grown on six well plates were transfected with Effectene reagents (Qiagen) according to the manufacturer's instructions. 500 ng of FIX exon 5 minigenes were transfected either alone or with 500 ng of ExSpeU1-encoding plasmids and the same was performed for co-transfection of splicing factors as previously reported [28, 40, 41]. GFP expression was routinely assessed in cotransfection experiments and showed more than 80% of transfection efficiency. Cells were incubated for 24 hours and then collected for RNA analysis. Total RNA extraction was performed using TRIreagent (Invitrogen) and cDNA was generated using 2 μg of total RNA and M-MuLV Reverse Transcriptase (NEB, UK). Alpha2,3 and Bra2 oligonucleotides were used for PCR amplification of pTBFIX exon 5 minigenes, as described previously.[9] PCR products were resolved on 1.5% agarose gel electrophoresis. Quantification of exon inclusion was performed using the ImageJ software. Protein assays: Expression vectors for FIX exonic variants were transiently transfected in HEK293 cells, and secreted FIX antigen (ELISA) and coagulant activity (aPTT-based assays) were evaluated as previously described.[39]

### Identification of proteins bound to RNA: pull-down analysis and mass spectrometry

For the pull-down analysis, the RNA templates were short RNA oligonucleotides, listed in S2 Table and the protocol was previously described.[29] Briefly, 10 μg of RNA oligo treated with m-periodate were mixed with dehydrazide agarose beads (Sigma) equilibrated with NaOAc and incubated on a rotator at 4°C overnight. After washing with Solution D (20 mM Hepes pH = 7.9, 100 mM KCl, 0.2 mM EDTA pH = 8.0, 100 mM DTT, 6% v/v Glycerol), the RNA-beads complex was incubated with HeLa cell nuclear extract (C4, Biotech) and 6 mg/mL of heparin. The beads were then washed six times with Solution D and the samples were loaded on 12% SDS-polyacrylamide gels. Gels were stained with Coomassie brilliant blue R250. The protein bands were excised and analyzed with mass spectrometer (LCQ DECA XP-ThermoFinnigam) and proteins were identified by analysis of the peptide MS/MS data with Turbo SEQUEST (Thermo Finnigam, CA, USA) and MASCOT (Matrix Science, UK). For the validation, protein samples were separated by NuPAGE 4%–12% Bis-Tris precast gels (Life Technologies, CA, USA) and electroblotted onto nitrocellulose membranes. The primary antibodies that were applied in western blotting analysis are: rabbit polyclonal anti-hnRNPA1 antibody (1:1000, Santa Cruz) and rabbit polyclonal anti-DAZAP1 antibody (1:1000).

### Small interfering (siRNA) transfection and U1 snRNA decoy

Silencing for *HNRNPA1/2*, *DAZAP1* and *SRSF2* was performed twice after 24 and 48 hours using Oligofectamine (Invitrogen, CA, USA), according to the manufacturer's instructions. The sense strands of RNAi oligos (Dharmacon, CO, USA and Sigma Aldrich, MO, USA), which were used to silence the target genes, are listed in the S3 Table. 24 hours after the second treatment with siRNA the cells were transfected with the minigenes, as described above. After additional 24 hours, cells were collected and divided in two equal fractions for RNA and protein depletion analysis. Confirmation of *HNRNPA1/2* and *DAZAP1* silencing was done using Western blot (antibodies listed above) and for the *SRSF2* using Sybr Green qPCR and ΔΔCt relative quantification with GAPDH as an endogenous control. The primers are listed in S4 Table. U1 snRNA 5′ functional inhibition was achieved by co-transfection of D1 plasmid as previously described.[27]

### In silico analysis of splicing factors

The *in silico* splicing analyses were performed using Human Splicing Finder (HSF)[23] with implementation of ESS Hexamers[25], predicted sites of SR proteins binding with ESE finder [22] and *SRSF2* consensus [26].

## Supporting Information

S1 Fig Restoration of exon skipping upon SRSF2 overexpression. Quantification and gel image of exon inclusion levels of FIX exon 5 10bp deletions co-trasfected with the SRSF2 overexpression plasmid.
(PDF)

S2 Fig Rescue of Δ2, Δ3, Δ7, Δ9 and Δ10 FIX exon 5 deletions by the ExSpeU1 FIX9. Minigenes with 10 bp deletions were co-transfected with ExSpeU1 FIX9. Splicing analysis of exon inclusion and exclusion levels shown complete rescue with the ExSpeU1 FIX9.
(PDF)

S3 Fig Identified FIX exon 5 RNA-bound proteins by mass-spectroscopy. Coomasie Blue stained SDS-PAGE gel showing the identification of proteins bound to RNA by mass spectroscopy.
(PDF)

S1 Table Oligonucleotides used for the cloning. (PDF)

S2 Table RNA oligos used for protein pull-down. (PDF)

S3 Table Sequences of the sense strand of RNAi oligonucleotides. (PDF)

S4 Table qPCR oligos. (PDF)
