# Supplementary figures and images for: Molecular Basis and Therapeutic Strategies to Rescue Factor IX Variants That Affect Splicing and Protein Function
Source: PLoS Genet. 2016 May 26;12(5):e1006082. doi: 10.1371/journal.pgen.1006082 (PMC4882169; doi:10.1371/journal.pgen.1006082)

Supplementary Figure 1

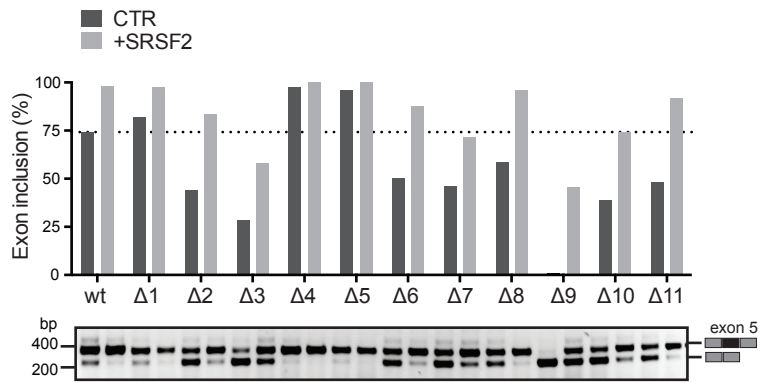

Supplement: S1 Fig — Quantification and gel image of exon inclusion levels of FIX exon 5 10bp deletions co-trasfected with the SRSF2 overexpression plasmid. (PDF) [file pgen.1006082.s001.pdf]

Supplementary Figure 2

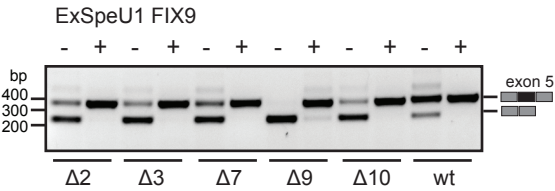

Supplement: S2 Fig — Minigenes with 10 bp deletions were co-transfected with ExSpeU1 FIX9. Splicing analysis of exon inclusion and exclusion levels shown complete rescue with the ExSpeU1 FIX9. (PDF) [file pgen.1006082.s002.pdf]

Supplementary Figure 3

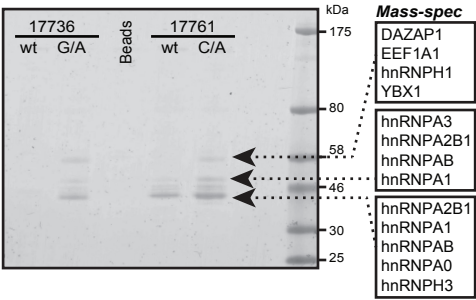

Supplement: S3 Fig — Coomasie Blue stained SDS-PAGE gel showing the identification of proteins bound to RNA by mass spectroscopy. (PDF) [file pgen.1006082.s003.pdf]
