# Supplementary material for: Molecular Basis and Therapeutic Strategies to Rescue Factor IX Variants That Affect Splicing and Protein Function
Source: PLoS Genet. 2016 May 26;12(5):e1006082. doi: 10.1371/journal.pgen.1006082 (PMC4882169; doi:10.1371/journal.pgen.1006082)
Supplement: S1 Table — (PDF) [file pgen.1006082.s004.pdf]

Suppl. Table 1 Oligonucleotides used for the cloning.

Plasmid	Name of oligonucleotide	5'-3' sequence
FIX6	FIX6_F	gatctcATAAAATCTTATTCAGATTgcaggggagataccat
	FIX6_R	gatcatggtatctcccctgcAATCTGAATAAGATTTT atga
FIX7a	FIX7a_F	gatctcATAAAAAAATCTTATTCAGATgcaggggagataccat
	FIX7a_R	gatcatggtatctcccctgcATCTGAATAAGATTTTTTTatga
FIX7b	FIX7b_F	gatctcATCTTATTCAGATgcaggggagataccat
	FIX7b_R	gatcatggtatctcccctgcATCTGAATAAGatga
FIX9	FIX9_F	gatctcATTCTTATTCAGgcaggggagataccat
	FIX9_R	gatcatggtatctcccctgcCTGAATAAGAatga
FIX13	FIX13_F	gatctcARAAAATCTTATgcaggggagataccat
	FIX13_R	gatcatggtatctcccctgcATAAGATTTTatga
FIX16	FIX16_F	gatctcTATAAAAAAATCTgcaggggagataccat
	FIX16_R	gatcatggtatctcccctgcAGATTTTTTATatga
FIX22	FIX22_F	GatctcATATTTCTTAAGgcaggggagataccat
	FIX22_R	gatcatggtatctcccctgcTTTAAAGAATatga
FIX33	FIX33_F	gatctcATTCAGATACAGAgcaggggagataccat
	FIX33_R	gatcatggtatctcccctgcTCTGTATCTGAatga
FIX38	FIX38_F	gatctcATAGTTTCAGATgcaggggagataccat
	FIX38_R	gatcatggtatctcccctgcATCTGAAACTatga
Q97E	C17704G_F	AGgAGTTTTGTAAAAATAGTGCTGATAACAAGGTGG
	C17704G_R	CAAAACTcCTCGCATCTGCCATTCTTAA
Q97K	C17704A_F	GGCAGATGCGAGRAGTTTTGTAAAAATAGTGCTG
	C17704A_R	CAGCACTATTTTTACAAAACTYCTCGCATCTGCC
Q97Stop	C17704T_F2	CAGATGCGAGtAGTTTTGTAAAAATAGTGCTGATAACAAGGTG
	C17704T_R2	TTACAAAACTaCTCGCATCTGCCATTCTTAATG
V107V	G17736A_F	GCTGATAACAAGGTAGTTTGCTCCTGTAC
	G17736A_R	GTACAGGAGCAAACTACCTTGTTATCAGC
G118E	G17756A_F	GCTCCTGTACTGAGGAATATCGACTTGCAG
	G17756A_R	CTGCAAGTCGATATTCCTCAGTACAGGAGC
G118A	G17756C_F	GCTCCTGTACTGAGGCATATCGACTTGCAG
	G17756C_R	CTGCAAGTCGATATGCCTCAGTACAGGAGC
G118V	G17756T_F	GCTCCTGTACTGAGGTATATCGACTTGCAG
	G17756T_R	CTGCAAGTCGATATACCTCAGTACAGGAGC
R116R	C17761A_F	CTGTACTGAGGGATATAGACTTGCAGAAAA
	C17761A_R	GTTTTCTGCAAGTCTATATCCCTCAGTACAG
R116G	C17761G_F	CTGTACTGAGGGATATGGACTTGCAGAAAAC
	C17761G_R	GTTTTCTGCAAGTCCATATCCCTCAGTACAG
R116Stop	C17761T_F	CTGTACTGAGGGATATTGACTTGCAGAAAAC
	C17761T_R	GTTTTCTGCAAGTCAATATCCCTCAGTACAG
L117F	C17764T_F	CTGAGGGATATCGATTTGCAGAAAACCAGAAG
	C17764T_R	CTTCTGGTTTTCTGCAAATCGATATCCCTCAG
A118V	C17768T_F	GGGATATCGACTTGTAGAAAACCAGAAGTC
	C17768T_R	GACTTCTGGTTTTCTACAAGTCGATATCCC
N120Y	A17773T_F	GATATCGACTTGCAGAATACCAGAAGTCCTGTG
	A17773T_R	CACAGGACTTCTGGTATTCTGCAAGTCGATATC
Q121Stop	C17776T_F	GACTTGCAGAAAACTAGAAGTCCTGTGAAC
	C17776T_R	GTTCACAGGACTTCTAGTTTTCTGCAAGTC
Q121H	G17778T_F	CTTGCAGAAAACCATAAGTCCTGTGAACCAG
	G17778T_R	CTGGTTCACAGGACTTATGGTTTTCTGCAAG
S123P	T17782C_F	CAGAAAACCAGAAGCCCTGTGAACCAGCAG
	T17782C_R	CTGCTGGTTCACAGGGCTTCTGGTTTTCTG
S123C	C17783G_F	CAGAAAACCAGAAGTGCTGTGAACCAGCAGG
	C17783G_R	CCTGCTGGTTCACAGCACTTCTGGTTTTCTG
