# Supplementary material for: Molecular Basis and Therapeutic Strategies to Rescue Factor IX Variants That Affect Splicing and Protein Function
Source: PLoS Genet. 2016 May 26;12(5):e1006082. doi: 10.1371/journal.pgen.1006082 (PMC4882169; doi:10.1371/journal.pgen.1006082)
Supplement: S2 Table — (PDF) [file pgen.1006082.s005.pdf]

Suppl. Table 2 RNA oligos used for protein pull-down.

Name of oligonucleotide	5'-3' sequence
FIX_17736 wt	AUAACAAGGUGGUUUGCUCCU
FIX_17736 G/A	AUAACAAGGUAGUUUGCUCCU
FIX_17761 wt	AGGGAUAUCGACUUGCAGAAA
FIX_17761 C/A	AGGGAUAUAGACUUGCAGAAA
