# Supplementary material for: Molecular Basis and Therapeutic Strategies to Rescue Factor IX Variants That Affect Splicing and Protein Function
Source: PLoS Genet. 2016 May 26;12(5):e1006082. doi: 10.1371/journal.pgen.1006082 (PMC4882169; doi:10.1371/journal.pgen.1006082)
Supplement: S3 Table — (PDF) [file pgen.1006082.s006.pdf]

Suppl. Table 3 Sequences of the sense strand of RNAi oligonucleotides.

Target gene	5’-3’ sequence
hnRNPA1	CAGCUGAGGAAGCUCUUCA
hnRNPA2	GGAACAGUUCCGUAAGCUC
DAZAP1	GAGACUCUGCGCAGCUACU
SRSF2	AAUCCAGGUCGCGAUCGAA
Luciferase	GCCAUUCUAUCCUCUAGAGGAUG
