# Supplementary material for: Molecular Basis and Therapeutic Strategies to Rescue Factor IX Variants That Affect Splicing and Protein Function
Source: PLoS Genet. 2016 May 26;12(5):e1006082. doi: 10.1371/journal.pgen.1006082 (PMC4882169; doi:10.1371/journal.pgen.1006082)
Supplement: S4 Table — (PDF) [file pgen.1006082.s007.pdf]

Suppl. Table 4 qPCR oligos

Name of oligonucleotide	5'-3' sequence
SRSF2_F	GCCGCAGCCGATCC
SRSF2_R	ACGAGGACTTGGACTTGG
GAPDH_F	GACAGTCAGCCGCATCTTCT
GAPDH_R	TTAAAAGCAGCCCTGGTGAC
